# Combining Quantitative and Qualitative Data in the Study of Feeding Behavior in Male Wistar Rats

**DOI:** 10.3389/fpsyg.2019.00881

**Published:** 2019-04-24

**Authors:** Maurizio Casarrubea, Stefania Aiello, Giuseppe Di Giovanni, Andrea Santangelo, Manfredi Palacino, Giuseppe Crescimanno

**Affiliations:** ^1^Laboratory of Behavioral Physiology, Department of Biomedicine, Neuroscience and Advanced Diagnostics, Human Physiology Section “Giuseppe Pagano,” University of Palermo, Palermo, Italy; ^2^Laboratory of Neurophysiology, Department of Physiology and Biochemistry, Faculty of Medicine and Surgery, University of Malta, Msida, Malta; ^3^Psychiatric Unit, Department of Health Sciences, University of Florence, Florence, Italy

**Keywords:** feeding behavior, standard diet, hyperglycidic diet, T-pattern analysis, TPA, rat

## Abstract

The first step in a behavioral study is represented by the organization of a suitable ethogram, that is, a formal description of individual components of the behavior. Then, each component of such a behavioral repertoire can be *quantified* (e.g., how many times it occurs, its duration, percent distribution, latency, etc.). However, it is our contention that the possibility to describe the behavior of a living being by means of hundreds or even thousands of numbers concerning isolated components, disjointed from the comprehensive behavioral architecture, does not imply the possibility to use those numbers to reconstruct the meaning of behavior. Such a level of comprehension requires a *qualitative approach* based on the analysis of behavioral structure and its underlying dynamics. By means of synergic utilization of quantitative and qualitative data a more complete description of a given behavior becomes available. In present study we discuss results obtained from observations of feeding behavior in two groups of male Wistar rats: a control group, under standard diet, and a second group, under hyperglycidic one. Results have been presented both in terms of quantitative evaluations and in terms of structural/qualitative ones, the latter obtained by means of T-pattern detection and analysis. As to quantitative results, mean durations showed a significant reduction of Walking and Feeding and an increase of Hind-Paw Licking and Body Grooming; concerning mean occurrences, a significant increase of Front-Paw Licking, Hind-Paw Licking, and Body Grooming was present; percent distributions showed significant reductions for Walking and Feeding and a significant increase for all grooming activities. As to qualitative assessments, T-pattern analysis unveiled a clear-cut behavioral reorganization induced by the hyperglycidic diet. If on the one hand, 50 different T-patterns were detected in subjects under standard diet, on the other hand, 703 different T-patterns were discovered in animals under hyperglycidic treatment, with a highly significant increase of mean lengths and a significant reduction of mean occurrences of T-patterns. Synergic evaluation of results in terms of quantitative and qualitative aspects shows, in rats fed with hyperglycidic diet, an increased anxiety condition, likely dependent on food-related stimuli and suggestive of a pervasive *craving-*related behavior.

## Introduction

The word “*diet*” comes from the ancient Greek δíαιτα, meaning “*way of life.*” Such a simple notion of etymological order is enough to understand the crucial role of nutrition in our daily life. However, human approach to nutrition, over the centuries, has moved from simple aspects related to survival to the point of representing, today, an instrument of personal satisfaction, often leading to unhealthy habits. When a person follows an incorrect diet it means that his food intake is not balanced, with some nutrients lacking, and others in excess. Examples in this sense abound. It is sufficient, for instance, to think of the typical menu of modern fast-food restaurants, extremely rich in salts, sugars, fats and, consequently, calories. The World Health Organization [WHO] (2018) clearly underlines how, today, there are almost 2 billion adults in the world, over the age of 18 or over, overweight. Of these, more than 600 million are obese or severely obese. The situation is even more dramatic if these already large numbers are added to the 38 million children under 5 years old who are overweight or obese (World Health Organization [WHO], 2018). Unfortunately, the problem of overweight and/or obesity is not only aesthetic. It is very well known, indeed, that an unhealthy diet increases the risk of developing numerous diseases such as those affecting cardiovascular, endocrine and osteoarticular systems; notably, a significant correlation between the intake of specific foods and the onset of certain types of cancer has been suggested as well (Scarborough et al., 2011; World Health Organization [WHO], 2018). It goes without saying that the social economic-burden related to diseases resulting from an unhealthy diet is, simply stated, incalculable (Scarborough et al., 2011). A problem studied with increasing attention only in relatively recent times concerns the relationships between addiction and nutrition. Food addictions, just like those related to substances of abuse, are dramatically common and ever-increasing (O’Brien, 2003; Sobik et al., 2005). In this context carbohydrates have a particularly important position. Nutritionists know well, concerning humans, that a hyperglycidic diet can neither be proposed nor tolerated because it has devastating effects on the body’s insulin levels with serious effects, in the long run, involving various organs and systems, including the central nervous system. Carbohydrates, taken indiscriminately and excessively, induce a perennial hunger that forces them to eat continuously, in a real state of metabolic dependence (Braga, 2010). Beyond the purely metabolic aspects, even from the behavioral point of view, an unbalanced high-carbohydrate diet induces important behavioral changes that have the connotations of a *real addiction*. This is true not only for human beings, but also it has been demonstrated in rats (Grimm et al., 2007). Aim of the present paper is twofold: first, to compare possible differences between rats under normal and hyperglycidic diet; second, to show the usefulness of a synergic utilization of quantitative and qualitative analyses in providing a more complete description of the studied behavior. To these purposes, sixteen male Wistar rats, divided into two groups, fed with two different diets (standard and hyperglycidic) and tested in an open field (OF) with free access to food and water, were analyzed. As to the above-mentioned joint use of quantitative and qualitative assessments, it is important to underline that, by definition, the so-called “mixed methods” (Anguera et al., 2018) refer to a synergic utilization of both quantitative and qualitative data, in the same research project, aimed at describing a given phenomenon in a more comprehensive way (Onwuegbuzie and Leech, 2010; Anguera et al., 2018). The utilization of these approaches orbits around the study of human behavior (Johnson et al., 2007). Of course, when animal behavior is analyzed the situation radically changes. In the following section, a brief overview of our perspective on this subject will be presented.

## Materials and Methods

### Quantitative and Qualitative Approaches in the Study of Rat Behavior

**FIGURE 1:**
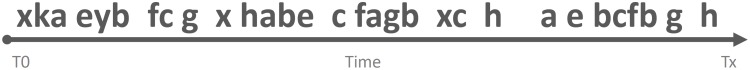
Short string of 30 hypothetical behavioral components (letters) occurring during a given T0–Tx time window (*X*-axis). Two hidden sequences are present. Even so, albeit the amount of components is small, the detection of such sequences is an extremely difficult task.

The behavior of a living being is structured on the basis of events flowing in time. The first and most obvious question of the researcher in the field of behavioral sciences, in both humans and non-humans, is how to study these events. Figure 1 represents a series of hypothetical behaviors during a given observation time window. In this figure, a first and intuitive assessment is to count the number of behaviors, thirty in this example; it follows the possibility to build a distribution of occurrences, then a percent distribution for each behavioral component etc. The quantification of the observed phenomena, in this case, a string of events on an axis, is an intuitive step that the researcher in the field of behavioral sciences performs and, more generally, it is a typically human approach to interface with reality. Imagine a table with scattered pencils on it: the first and most obvious evaluation is the numerical one. How many? Then, you could count how many of a color and how many of another color etc. How many of us, seeing objects on a table, would consider how do they relate to each other?

**FIGURE 2:**
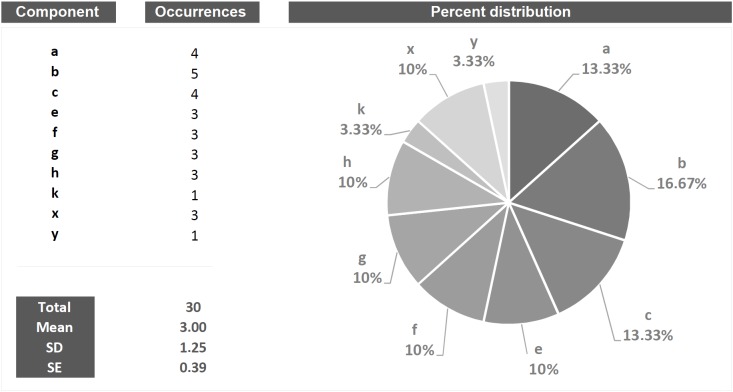
Example of quantitative approach to the analysis applied to the string of 30 hypothetical components showed in Figure 1. Columns on the left show all the components in terms of their occurrences, total occurrences, mean occurrences, SD and SE. The pie chart shows the percent distribution of each component.

*Quantitative* is that approach which, by its own nature, provides numerical measures concerning the object studied (Onwuegbuzie and Leech, 2010; Casarrubea et al., 2017c). In behavioral terms, a purely quantitative approach will be able to answer important questions concerning, for example, how many behaviors of a given type occur, their duration, their percent distribution, which behavior is more frequent, which the least frequent etc. Figure 2 presents, in quantitative terms, the string of events illustrated in Figure 1. Undoubtedly, these numbers provide the reader with a great sense of exhaustiveness. However, it should be noted that a characteristic of the data shown in Figure 2 is the lack of the slightest information inherent possible relationships between the various events. In other words, these quantities describe behaviors isolated from each other, separated from what is, actually, the real behavioral architecture and its intrinsic qualities. *This is not different from classifying all the single pieces of a puzzle missing the comprehensive picture*. The functional meaning of a behavior, i.e., the study of the existing interplay between an animal and the context, is a picture lying in its intrinsic structural features.

**FIGURE 3:**
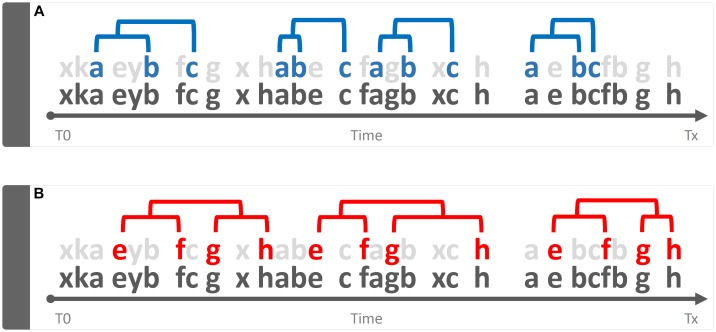
Example of a qualitative approach to the analysis of the string of 30 hypothetical components showed in Figure 1. Bottom strings in both panels represent the same sequence illustrated in Figure 1 by removing “background noise” events, two sequences a-b-c (panel **A**) and e-f-g-h (panel **B**) become evident. Concerning quality, three different issues can be mentioned here namely: *Complexity* (sequence in **B** more complex than **A**), *Recursivity* (sequence in **A** more recursive than **B**) and *Variability* (when two or more subjects are compared, the behavior of one can be considered more variable if more different sequences are performed).

*Qualitative* is that approach that tends to magnify the properties of the investigated object by studying “phenomena in their natural setting” (Onwuegbuzie and Leech, 2010). In behavioral terms, a qualitative approach is able to shed light on aspects such as, for example, the greater or lesser *complexity* of a behavior, its *variability*, its *coherence* in accordance with the context, etc. Taking into consideration Figure 3, the bottom line of both panels is identical to that shown in Figure 1. However, two sequences (a-b-c) and (e-f-g-h) are present and well appreciable only if the “background noise” of the other elements is removed or, in any case, reduced (Figure 3, gray letters). This very simple example raises an equally simple question: what is the meaning of these recurring patterns? The simplest way to answer this question is: emerging phenomena characterized, as such, by qualitative aspects that cannot be inferred from the single structural elements that compose them. These structures, called “T-patterns,” find their *raison d’être* in the distances between the various events in sequence: time distances, statistically evaluated, which make each of these sequences relatively self-similar (Casarrubea et al., 2015; Magnusson, 2016). A more detailed description of T-pattern detection process will be presented in Section “Data Analysis”. Concerning above-mentioned qualitative features, various aspects could be highlighted. First, even at a very first glance it appears evident that T-pattern in panel B does contain more events in sequence than T-pattern in panel A. Hence, it is possible to state that the four events T-pattern on the bottom is more *complex*. On the contrary, T-pattern in panel A does occur more often than T-pattern in B. Thus, behind this perspective, it is possible to state that T-pattern A is more *recursive*. Finally, when two or more subjects are compared, the behavior of one can be considered more *variable* depending on the number of different T-patterns performed (for example, subject #1 presents 5 different T-patterns, subject #2 presents 10 different T-patterns). It is important to underline that the three qualities above mentioned, namely, complexity, recursivity and variability have meanings only if *relationships among events* in sequence are considered because, simply stated, they do arise from *structural* features of the behavior.

### Animals and Housing

Sixteen male Wistar rats (Harlan Laboratories, Italy) were used. At their arrival, all rats were fed for 1 week with standard (55% carbohydrates) pellets (Mucedola, Italy). Then, animals were randomly divided into two groups, each group encompassing eight subjects. Standard laboratory pellets and water were freely accessible for the first group, used as a control; hyperglycidic (70% carbohydrates) pellets (Mucedola, Italy) and water were freely accessible for the second group. All subjects were housed in a room maintained at 23 ± 1°C with the light on 07:00 a.m. and off 07:00 p.m. They were tested, after 1 month of standard or hyperglycidic diet, when they were 2 months old.

### Experimental Apparatus

**FIGURE 4:**
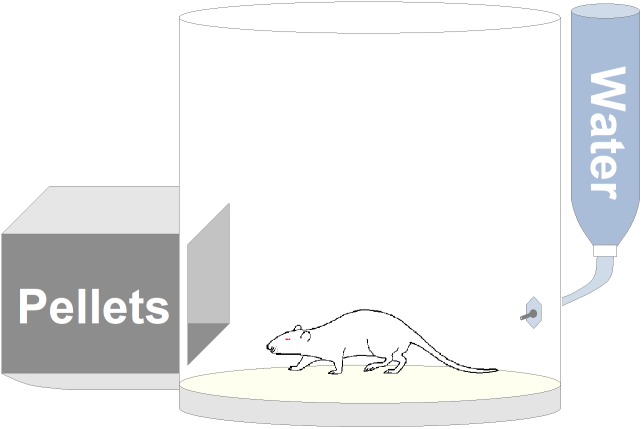
Outline of the experimental apparatus.

Apparatus consisted of a circular (ϕ 35 cm) open-field (OF) arena made of white opaque Plexiglas with two openings, through which the rat could have free access to a pellet box and a spout for dispensing water. An outline of this experimental apparatus is presented in Figure 4. Animal’s behavior was recorded through a digital camera (Toshiba HD-DV camcorder P10) placed in front of the OF and video files stored in a personal computer for following analyses.

### Procedure

Animals were transported from the housing room to testing room within their home cages. In the testing room, to avoid possible visual and olfactory influences, all subjects were allowed to acclimate for 30 min far from observational apparatus. The temperature in the testing room, where the OF was placed, was maintained equal to the temperature in the housing room. Each animal, experimentally naïve, was placed in the OF, allowed to freely explore for 60 min and observed only once. After each observation, OF apparatus was carefully cleaned with ethyl alcohol (70%). Procedural details described in this section were carried out on the basis of our protocols (Casarrubea et al., 2011a,b, 2016b, 2017a,b).

### Data Analysis

The ethogram presented in Table 1 is based on behavioral categories previously employed (Casarrubea et al., 2009, 2011a, 2017b; Santangelo et al., 2017, 2018). It encompasses: Walking (Wa), climbing (Cl), immobile sniffing (IS), feeding (Fe), focused sniffing (FS), drinking (Dr), front-paw licking (FPL), hind-paw licking (HPL), face grooming (FG), body grooming (BG), and immobility (Im). The behavior of each subject was annotated by means of a software tool (The Observer, Noldus Information Technology bv, Netherlands) and event log files were generated for each subject. Event log files were then processed using Theme (PatternVision Ltd., Iceland), a computer program able to detect sequences of events on the basis of the existence of statistically significant constraints on the intervals separating them (Magnusson, 1996, 2000, 2016; Casarrubea et al., 2015). In brief, given a distribution of events occurring within a T0–Tx observation period (Figure 3A, bottom row events, near *X*-axis) the program compares the distributions of each pair of the behavioral events “a” and “b” searching for a time window so that “a” is followed by “b” within that time interval. If this condition between event “a” and event “b” does occur, a two-event first level T-pattern, namely (a b), is detected (Figure 3A, upper row events); in a second step such first level T-pattern is considered as “a” or “b” terms for the detection of higher order temporal patterns, e.g., ([a b] c)…and so on (Figure 3A, upper row events). A more detailed description of theories and concepts concerning T-pattern detection and analysis can be found in our previous articles (Casarrubea et al., 2013, 2016a, 2017a), in two reviews (Casarrubea et al., 2015, 2018) or in our book (Magnusson et al., 2016). On the basis of our previous studies (Casarrubea et al., 2011a,b, 2016b, 2017a,b) the following parameters were analyzed: (1) mean duration (in sec) of behavioral components; (2) mean occurrences of behavioral components; (3) percent distribution of behavioral components; (4) number of different T-patterns detected for each group both in real and random generated data; (5) length distribution of T-patterns; (6) mean length of T-patterns detected; (7) mean occurrences of T-patterns detected and (8) percent distribution of T-patterns including each components of the behavioral repertoire; (9) structure of all the different T-patterns detected for each group (strings).

**Table 1:** Ethogram of rat behavior.

Walking (Wa)	The rat walks around sniffing the environment
Climbing (Cl)	The rat maintains an erect posture leaning against the Plexiglas wall
Immobile Sniffing (IS)	The rat sniffs the environment firmly standing on the ground
Feeding (Fe)	The rat eats from an apposite pellet-box
Focused Sniffing (FS)	The rat sniffs the border of the feeding pellet-box without inserting the head inside
Drinking (Dr)	The rat drinks from an apposite water-pipe
Front Paw Licking (FPL)	The rat licks or grooms its forepaws
Hind Paw Licking (HPL)	The rat licks or grooms its hind paws
Face Grooming (FG)	The rat rubs its face with the forepaws
Body Grooming (BG)	The rat rubs the body combing the fur by fast movement of the incisors
Immobility (Im)	The rat maintains a fixed posture

### Statistics

As to quantitative evaluations, possible significant outcomes concerning mean frequencies and mean durations between groups were assessed using Student’s *t*-test for independent samples; percent distribution of behavioral components in both groups were compared used Chi-square test. As to T-pattern analysis, albeit each detected sequence implies an underlying significant relationship among the events in pattern, in data with thousands of events an exceptionally high number of possible relationships exists. Such an aspect could raise the issue of whether the T-patterns are detected only by chance. Theme deals with such an important issue by repeatedly randomizing and reanalyzing the original data, using the same search parameters utilized in the detection process carried out in the real data. Then, the mean number of T-patterns of each length identified in the randomized data is compared with that obtained from the original data. Mean length and mean occurrences of T-patterns between the two groups were compared using Student’s *t*-test. Finally, the percent distribution of T-patterns containing each component of the behavioral repertoire was compared using the Chi-square test.

### Ethics Statement

All efforts were made to minimize the number of animals used and their suffering. Experimental procedures were conducted in accordance with the European Communities Council Directive (2010/63/EU) and approved by the official Veterinary Committee appointed by the University of Palermo.

## Results

### Quantitative Results

**FIGURE 5:**
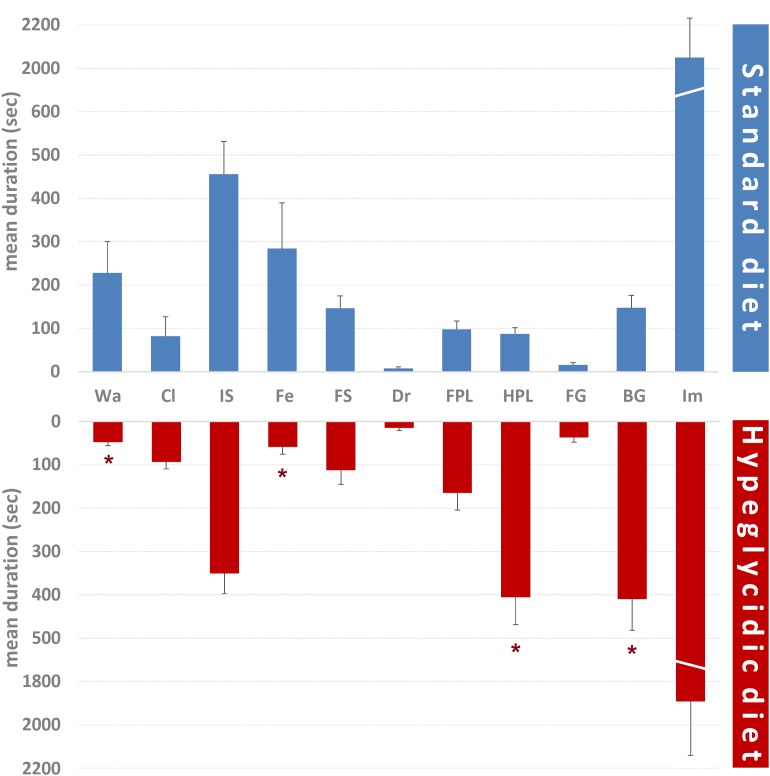
Mean duration ± SE (in s) of each component of the behavioral repertoire in rats under standard and hyperglycidic diet. Difference between standard and hyperglycidic group, as revealed by Student’s *t*-test for independent samples, ^∗^*p* < 0.05. Wa, walking; Cl, climbing; IS, immobile sniffing; Fe, feeding; FS, focused sniffing; Dr, drinking; FPL, front-paw licking; HPL, hind-paw-licking; FG, face grooming; BG, body grooming; Im, immobility. Data obtained from the analysis of sixteen subjects.

Mean durations of each component of the behavioral repertoire are represented in Figure 5. Comparison of subjects under standard vs. hyperglycidic diet showed significant changes for Wa (*t*_14_ = 2.468; *p* < 0.05), Fe (*t*_14_ = 2.206; *p* < 0.05), HPL (*t*_14_ = −4.941; *p* < 0.0001), and BG (*t*_14_ = −3.375; *p* < 0.005). Remaining components were: Cl (*t*_14_ = −0.237; *p* = 0.816), IS (*t*_14_ = 1.184; *p* = 0.256), FS (*t*_14_ = 0.779; *p* = 0.449), Dr (*t*_14_ = −1.066; *p* = 0.304), FPL (*t*_14_ = −1.534; *p* = 0.147), FG (*t*_14_ = −1.737; *p* = 0.104), and Im (*t*_14_ = 0.430; *p* = 0.674).

**FIGURE 6:**
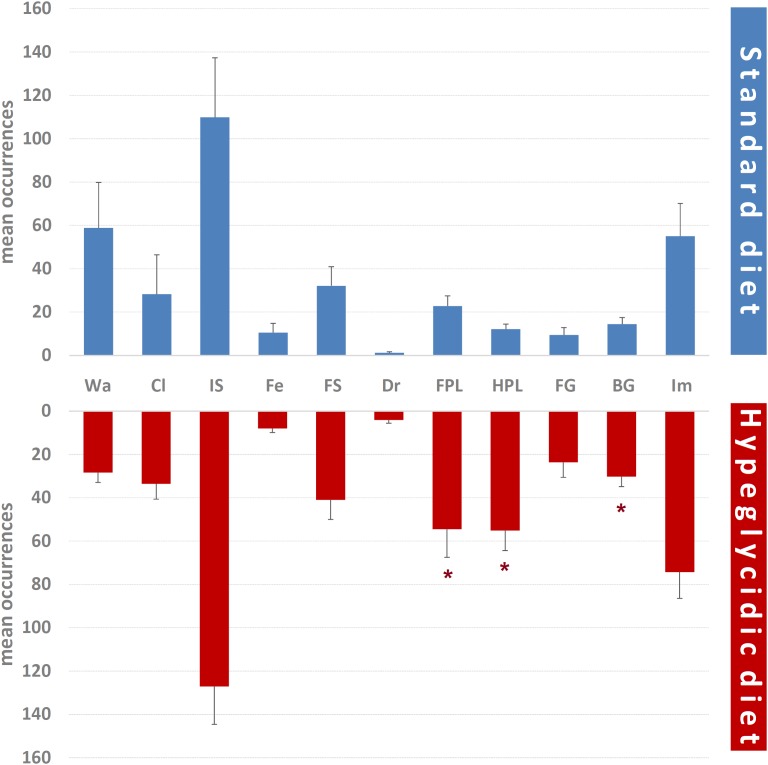
Mean occurrences ± SE of each component of the behavioral repertoire in rats under standard and hyperglycidic diet. ^∗^*p* < 0.05 difference between standard and hyperglycidic group, as revealed by Student’s *t*-test for independent samples. Wa, walking; Cl, climbing; IS, immobile sniffing; Fe, feeding; FS, focused sniffing; Dr, drinking; FPL, front-paw licking; HPL, hind-paw-licking; FG, face grooming; BG, body grooming; Im, immobility. Data obtained from the analysis of sixteen subjects.

Mean occurrences of each component of the behavioral repertoire are represented in Figure 6. Significant differences were detected for FPL (*t*_14_ = −2.301; *p* < 0.05), HPL (*t*_14_ = −4.520; *p* < 0.0001), and BG (*t*_14_ = −2.847; *p* < 0.05). Remaining components were: Wa (*t*_14_ = 1.409; *p* = 0.181), Cl (*t*_14_ = −0.269; *p* = 0.792), IS (*t*_14_ = −0.530; *p* = 0.604), Fe (*t*_14_ = 0.537; *p* = 0.61), FS (*t*_14_ = −0.706; *p* = 0.492), Dr (*t*_14_ = −1.959; *p* = 0.07), FG (*t*_14_ = −1.837; *p* = 0.088), and Im (*t*_14_ = −0.986; *p* = 0.341).

**FIGURE 7:**
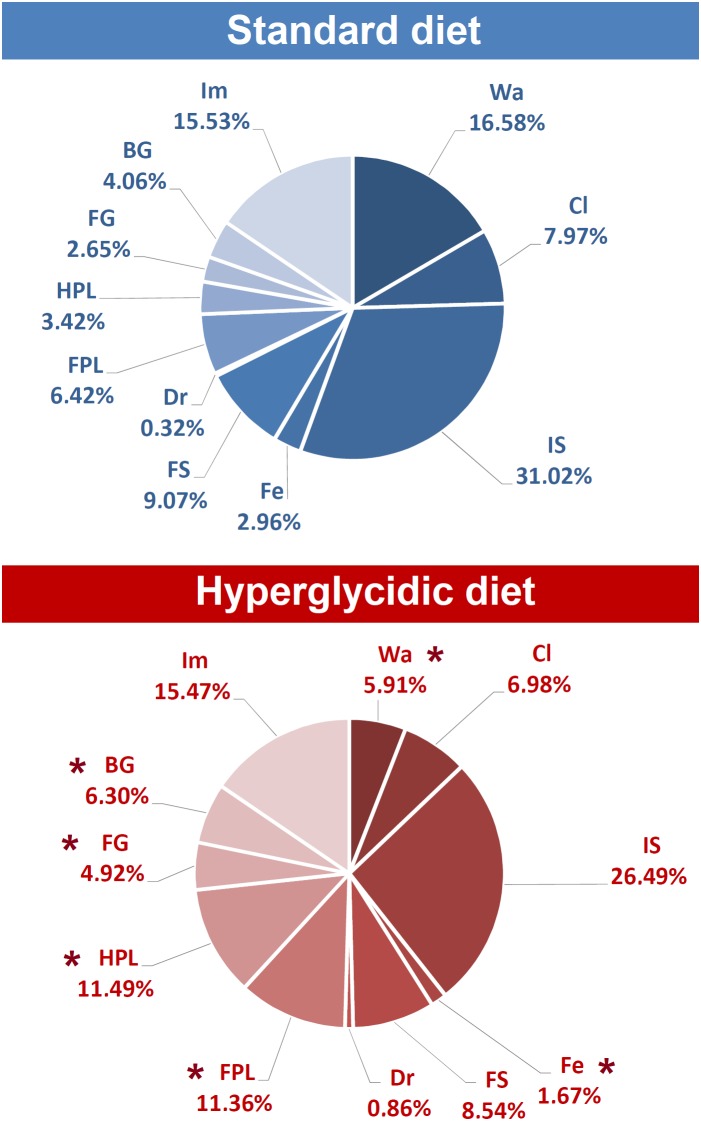
Percent distribution of each component of the behavioral repertoire in rats under standard and hyperglycidic diet. ^∗^*p* < 0.05 difference between standard and hyperglycidic group, as revealed by the Chi-square test. Data obtained from the analysis of sixteen subjects. Wa, walking; Cl, climbing; IS, immobile sniffing; Fe, feeding; FS, focused sniffing; Dr, drinking; FPL, front-paw licking; HPL, hind-paw-licking; FG, face grooming; BG, body grooming; Im, immobility. Data obtained from the analysis of sixteen subjects.

Percent distributions of behavioral components are represented in Figure 7. Chi-square test revealed significant reductions for Wa (*p* < 0.0001) and Fe (*p* < 0.005); significant increases have been detected for FPL (*p* < 0.005), HPL (*p* < 0.0001), FG (*p* < 0.005), and BG (*p* < 0.0001).

### Temporal Patterns of Behavior

**FIGURE 8:**
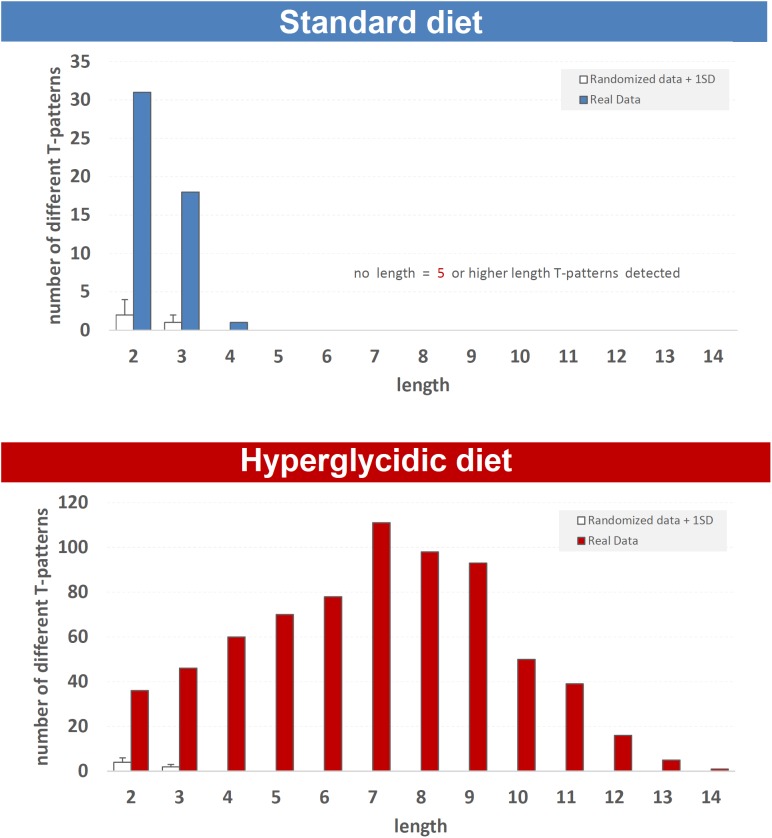
T-pattern length distribution in real data (filled bars) and randomized data + 1SD (white bars) in standard and hyperglycidic groups. *X*-axis = T-pattern length i.e., number of events in T-pattern’s structure; *Y*-axis = number of different T-patterns detected. Wa, walking; Cl, climbing; IS, immobile sniffing; Fe, feeding; FS, focused sniffing; Dr, drinking; FPL, front-paw licking; HPL, hind-paw-licking; FG, face grooming; BG, body grooming; Im, immobility. Data obtained from the analysis of sixteen subjects.

Fifty different T-patterns were detected in subjects under standard diet, and 703 in animals under hyperglycidic diet. On the basis of their length, T-patterns in both groups were distributed as follows. In the standard diet group, 31 different T-patterns encompass two events, 18 three events and 1 four events (Figure 8, upper panel). In subjects under hyperglycidic treatment, 36 T-patterns contain two events, 46 three, 60 four, 70 five, 78 six, 111 seven, 98 eight, 93 nine, 50 ten, 39 eleven, 16 twelve, 5 thirteen and, finally, 1 T-pattern has fourteen events in sequence (Figure 8, bottom panel). The comparison between the mean number of T-patterns detected in random data (mean value obtained on the basis of *n* = 5 random runs) vs. real data showed a significantly higher number of T-patterns in real data (Figure 8, white bars).

**FIGURE 9:**
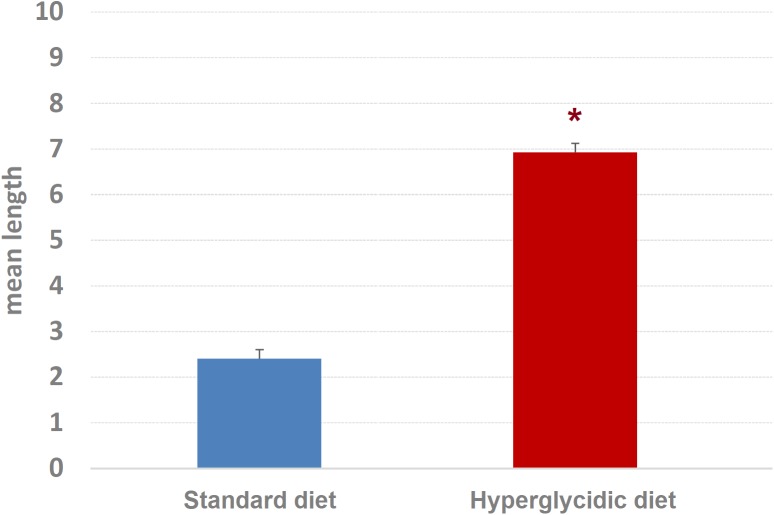
Mean length ± SE of T-patterns detected. Difference between standard and hyperglycidic group, as revealed by Student’s *t*-test for independent samples, ^∗^*p* < 0.05. Data obtained from the analysis of sixteen subjects.

**FIGURE 10:**
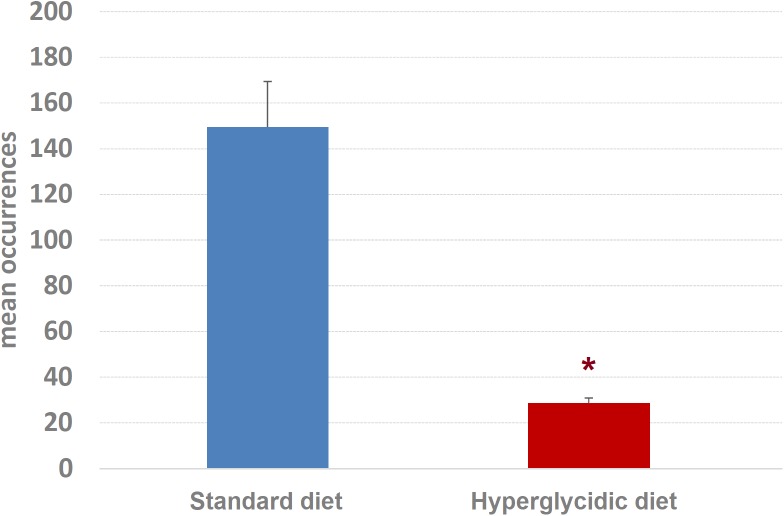
Mean occurrences ± SE of T-patterns detected. Difference between standard and hyperglycidic group, as revealed by Student’s *t*-test for independent samples, ^∗^*p* < 0.05. Data obtained from the analysis of sixteen subjects.

Concerning mean length of T-patterns, a highly significant increase was detected in hyperglycidic group, in comparison with the standard one (*t*_751_ = 12.502, *p* < 0.0001) (Figure 9); concerning mean occurrences of T-patterns, a highly significant reduction was detected (*t*_751_ = −12.399, *p* < 0.0001) (Figure 10).

Overall, in subjects under standard diet, the 50 different T-patterns occurred 7475 times, while in rats under hyperglycidic diet the 703 different T-patterns occurred 20148 times. In the Supplementary Table S1, the complete results of T-pattern detection for both groups are shown.

**FIGURE 11:**
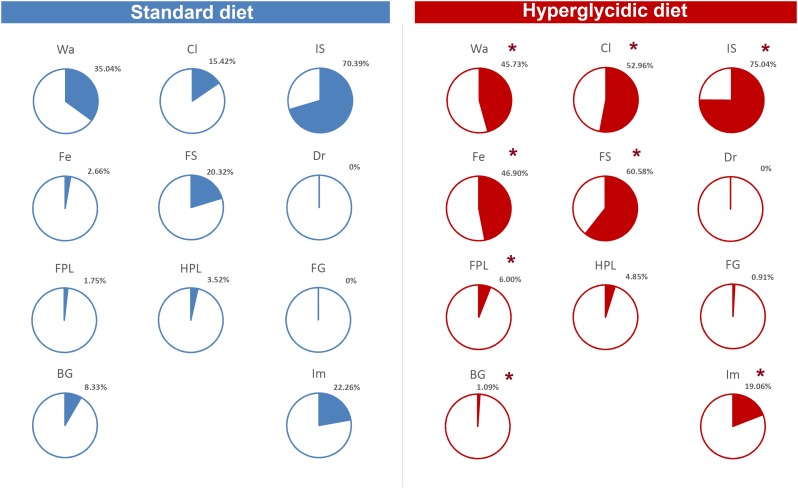
Percent distribution of T-patterns containing each element of the behavioral repertoire (see Table 1). To avoid exceedingly thin slices, percent values <1% were represented together (“Others”). Chi-square test, ^∗^*p* < 0.0001. Wa, walking; Cl, climbing; IS, immobile sniffing; Fe, feeding; FS, focused sniffing; Dr, drinking; FPL, front-paw licking; HPL, hind-paw-licking; FG, face grooming; BG, body grooming; Im, immobility. Data obtained from the analysis of sixteen subjects.

Finally, as to per cent of T-patterns containing at least one time each component of the behavioral repertoire in sequence, Chi-square test revealed highly significant (*p* < 0.0001) differences between the two groups, for T-patterns containing Wa, Cl, IS, Fe, FS, FPL, BG, and Im (Figure 11).

## Discussion

Behavioral studies, during the last decades, have been somewhat conservative in the renewal of their approaches. In fact, even a simple search through a common scientific database, with thousands of published papers so far, will show how purely quantitative evaluations have been, by far, preferred in data analysis and description of the results. The aim of this work is to show how, through the synergistic use of a quantitative and qualitative approach, it is possible to appreciate a much more complete portrayal of behavior and make available points of view and facets otherwise not deducible from the use of a purely qualitative or purely quantitative approach.

### Quantitative Assessments

Overall, all data concerning quantitative assessments (Figure 5–7), e.g., durations, frequencies and percent distributions show that the hyperglycidic diet induces an important change especially in grooming-related behaviors, i.e., FPL, HPL, and BG. In addition, Wa and Fe were reduced both in terms of duration and of percent distribution in subjects treated with the hyperglycidic diet. These results find support in scientific literature as it is very well known that rats under hyperglycidic diet develop a metabolic syndrome associated with severe anxiety-like behavior (Reddy et al., 2016; Rebolledo-Solleiro et al., 2017). Consistently, changes in subject’s anxiety condition are known to be related with clear-cut changes in grooming behavior (Berridge et al., 2005; Kalueff and Tuohimaa, 2005; Veloso et al., 2016).

### T-Patterns, Structures and Qualities

A more in-depth analysis, based on the quality of behavior in the two groups (Figure 8–11 and Supplementary Table S1) shows numerous additional aspects, which together with quantitative data provide a more harmonic representation of the effects of an unbalanced diet on behavior, and more in general, differences between the two groups of animals.

The T-patterns analysis shows great differences between the two groups. First of all, these differences appear in terms of different patterns identified and their length: if on the one hand the animals fed with standard diet showed a total of 50 T-patterns the longest of which contained four events in sequence, the animals with hyperglycidic diet showed a hugely more variable and complex behavior with 703 different T-patterns, the longest of which had 14 events in sequence. Consistently, the average length of the T-patterns reflects these notable differences. In contrast, the average recurrence of each T-pattern is much higher in subjects with a standard diet. Briefly, subjects with a standard diet show a behavior characterized by fewer different sequences (less *variability*) which are repeated more times than what is observed in the hyperglycidic diet group (greater *recursivity*). On the contrary, subjects with a hyperglycidic diet show a behavior characterized by a greatly higher number of different T-patterns (greater *variability*) which are, in turn, repeated much lesser than observed for sequences of the control group (less *recursivity*). These results, altogether with what traced by quantitative observations, offer a considerably expanded portrait of the behavioral dynamics elicited by the chronic exposition to hyperglycidic diet. The already reported result concerning a higher anxiety-like state following the hyperglycidic diet in rodents (Reddy et al., 2016; Rebolledo-Solleiro et al., 2017) well fits with many of the complex behavioral changes observed in our sample. In this sense, an important contribution to the interpretation of these data comes from the evaluation of the composition of the T-patterns in the two groups (Figure 11). The likely most salient outcome is represented by the evidence that the components Fe and FS are structured in an enormously higher percent of T-patterns in animals with a hyperglycidic diet. It is also important to notice a percent of T-patterns including Wa, Cl, and IS considerably increased. These data would seem to be in contrast with evaluations of durations and frequencies, where a reduction in Wa, Fe, and FS would appear, even statistically significant as regards the duration of Wa and Fe. Actually, what likely happens is that in animals treated with hyperglycidic diet, the behaviors correlated with food intake become structured in a number of T-patterns much higher than what happens with the standard diet. In this context, it is particularly interesting to observe the highly significant increase not only of Fe, but also of FS, that is the sniffing behavior of the pellet box. Taken together, these results suggest an increased salience of food-related stimuli in rats under hyperglycidic diet. Such an aspect fuels a stimulating topic of discussion concerning a putative change in the motivational drive toward obtaining and consuming food. In addition, T-pattern analysis describes how changes in food-related behaviors (Fe and FS) affect also the structural interplay with behaviors not directly related to food such as Wa, Cl, and IS. Such behavioral dynamics strongly oriented toward food stimuli are suggestive of *craving-*related behaviors. In fact, rats fed on a hyperglycidic diet show a totally reorganized behavior, structured and aimed at the continuous search for food. Interestingly, in rats, numerous tangencies have been demonstrated between the nerve circuits that mediate the craving mechanisms for opioids and carbohydrates and, in fact, the administration of an antagonist for opioids, naloxone, in these animals has proved effective in reducing the craving for sucrose (Grimm et al., 2007). From a translational perspective, this is consistent with the well-known addiction that an unbalanced high-carbohydrate diet induces in humans (O’Brien, 2003; Sobik et al., 2005; Braga, 2010), as mentioned in the introduction section. A final consideration concerns the limitation of this study. It is important to consider, indeed, that present results show the effects induced only by a hyperglycidic diet and only in male rats. These two aspects fuel interesting topics of discussion concerning possible future researches. For instance, it would be interesting to evaluate the behavioral effects induced, in rodents, also by high-protein or high-fat diets. Another interesting aspect would be the comparison of effects induced by unbalanced diets in male and female subjects and/or to assess possible sex differences under the same dietetic regime. Finally, taking into consideration the clear cut impact that hyperglycidic diet produced in terms of behavioral changes in rats, it might be interesting to evaluate also possible differences between different strains.

## Conclusion

In the context of a behavioral study, conceptual and procedural differences underlying quantitative and qualitative approach could lead to a superficial conclusion placing them in contrast. Such a position should be avoided because through the synergistic use of qualitative and quantitative evaluations it is possible to study behavior in a much more complete way. Here we have demonstrated that the consensual evaluation of the quantitative and qualitative data allows obtaining an evaluation that neither of the two approaches, individually, is able to offer. Specifically, if on the one hand, the important modification of the duration and percent of Wa and of all grooming activities allows to formulate the hypothesis of an anxiety-related behavior, on the other hand, the evaluation of the T-patterns highlights how such an anxiety condition is, from the behavioral point of view, only the tip of an iceberg, largely submerged, consisting of the evident behavioral restructuring that the hyperglycidic diet imposes. Such a reorganization has the features of a behavior largely oriented, from a qualitative perspective, toward food-related stimuli, enormously more than what happens in animals exposed to a balanced standard diet.

## Author Contributions

All authors listed have made a substantial, direct and intellectual contribution to the work, and approved it for publication.

## Conflict of Interest Statement

The authors declare that the research was conducted in the absence of any commercial or financial relationships that could be construed as a potential conflict of interest. The handling Editor declared a past co-authorship with one of the authors MC.

## Supplementary Material

TABLE S1 Terminal strings (behavioral components in brackets) of the T-patterns detected in standard and hyperglycidic groups. Progressive numbers on the left of each string only indicate the corresponding string. Numbers on the right of each string indicate length (i.e., number of events in T-pattern’s structure) and overall occurrences (Occs). Wa, walking; Cl, climbing; IS, immobile sniffing; Fe, feeding; FS, focused sniffing; Dr, drinking; FPL, front-paw licking; HPL, hind-paw-licking; FG, face grooming; BG, body grooming; Im, immobility. Data obtained from the analysis of sixteen subjects.
