# Supplementary material for: Combining Quantitative and Qualitative Data in the Study of Feeding Behavior in Male Wistar Rats
Source: Front Psychol. 2019 Apr 24;10:881. doi: 10.3389/fpsyg.2019.00881 (PMC6491709; doi:10.3389/fpsyg.2019.00881)
Supplement: TABLE S1 — Terminal strings (behavioral components in brackets) of the T-patterns detected in standard and hyperglycidic groups. Progressive numbers on the left of each string only indicate the corresponding string. Numbers on the right of each string indicate length (i.e., number of events in T-pattern’s structure) and overall occurrences (Occs). Wa, walking; Cl, climbing; IS, immobile sniffing; Fe, feeding; FS, focused sniffing; Dr, drinking; FPL, front-paw licking; HPL, hind-paw-licking; FG, face grooming; BG, body grooming; Im, immobility. Data obtained from the analysis of sixteen subjects. [file Data_Sheet_1.PDF]

Standard Diet

#	Terminal String	Length	Occs
1	(bg bg)	2	75
2	(bg is)	2	81
3	(cl is)	2	186
4	(cl wa)	2	152
5	(fe fs)	2	37
6	(fpl bg)	2	59
7	(fs fe)	2	54
8	(fs fs)	2	202
9	(fs im)	2	94
10	(fs is)	2	206
11	(fs wa)	2	142
12	(hpl bg)	2	22
13	(hpl fs)	2	36
14	(hpl hpl)	2	42
15	(hpl im)	2	51
16	(hpl is)	2	69
17	(im im)	2	349
18	(im is)	2	185
19	(is bg)	2	69
20	(is cl)	2	179
21	(is fe)	2	63
22	(is fpl)	2	72
23	(is fs)	2	188
24	(is im)	2	359
25	(is is)	2	779
26	(is wa)	2	358
27	(wa bg)	2	43
28	(wa cl)	2	149
29	(wa im)	2	167
30	(wa is)	2	360
31	(wa wa)	2	401
32	(bg (is bg))	3	43
33	(fs (is fs))	3	149
34	(fs (wa cl))	3	66
35	(hpl (is fs))	3	22
36	(im (is im))	3	280
37	(is (cl is))	3	171
38	(wa (is wa))	3	310
39	((bg is) bg)	3	52
40	((bg is) fs)	3	31
41	((bg is) wa)	3	39
42	((bg is)(wa is))	4	35
43	((fs is) fs)	3	175
44	((fs wa) cl)	3	72
45	((im is) im)	3	179
46	((is bg) hpl)	3	21
47	((is bg) is)	3	53

48	((is cl) is)	3	178
49	((is fs) fe)	3	45
50	((wa is) wa)	3	325
		**Total**	**7475**

Hyperglycidic diet

#	Terminal String	Length	Occs
1	(bg bg)	2	219
2	(cl cl)	2	237
3	(cl fe)	2	45
4	(cl fpl)	2	79
5	(cl fs)	2	109
6	(cl is)	2	227
7	(cl wa)	2	107
8	(fe cl)	2	39
9	(fe fs)	2	54
10	(fe is)	2	44
11	(fe wa)	2	35
12	(fpl fg)	2	183
13	(fs cl)	2	95
14	(fs fe)	2	54
15	(fs fs)	2	285
16	(fs hpl)	2	40
17	(fs im)	2	154
18	(fs is)	2	253
19	(fs wa)	2	59
20	(hpl hpl)	2	399
21	(im im)	2	473
22	(im is)	2	341
23	(is cl)	2	221
24	(is fe)	2	54
25	(is fpl)	2	167
26	(is fs)	2	252
27	(is im)	2	410
28	(is is)	2	907
29	(is wa)	2	191
30	(wa cl)	2	101
31	(wa fe)	2	29
32	(wa fpl)	2	35
33	(wa fs)	2	79
34	(wa im)	2	69
35	(wa is)	2	192
36	(wa wa)	2	190
37	(cl (fe cl))	3	36
38	(fe (is cl))	3	37
39	(fe (is fs))	3	51
40	(fs (cl fe))	3	16
41	(fs (cl fs))	3	89

42	(fs (fe cl))	3	34
43	(fs (fe fs))	3	48
44	(fs (fe is))	3	35
45	(fs (fe wa))	3	27
46	(fs (is fe))	3	20
47	(fs (is fpl))	3	31
48	(fs (is fs))	3	200
49	(im (fs im))	3	63
50	(im (is fpl))	3	47
51	(im (is im))	3	282
52	(is (cl is))	3	216
53	(wa (cl wa))	3	88
54	(wa (fe wa))	3	21
55	(wa (fs hpl))	3	29
56	(wa (fs im))	3	30
57	(wa (fs wa))	3	34
58	(wa (is fe))	3	28
59	(wa (is wa))	3	157
60	((cl fe) cl)	3	39
61	((cl fe) is)	3	36
62	((cl fe)(is cl))	4	35
63	((cl fpl) is)	3	60
64	((cl fpl) wa)	3	39
65	((cl fpl)(is wa))	4	38
66	((cl fs) cl)	3	90
67	((cl fs) fe)	3	41
68	((cl fs)(fe cl))	4	33
69	((cl fs)(fe is))	4	31
70	((cl fs)(is cl))	4	74
71	((cl is) cl)	3	198
72	((cl is)(fe cl))	4	32
73	((cl is)(fe is))	4	30
74	((cl wa) cl)	3	89
75	((cl wa)(fs cl))	4	39
76	((cl wa)(fs im))	4	29
77	((fe is) cl)	3	33
78	((fe is) fs)	3	36
79	((fe is) wa)	3	32
80	((fe is)(wa cl))	4	28
81	((fe is)(wa fs))	4	28
82	((fe is)(wa im))	4	25
83	((fs cl) fs)	3	88
84	((fs cl)(is fs))	4	73
85	((fs cl)(is im))	4	26
86	((fs cl)(wa fs))	4	47
87	((fs fe) is)	3	33
88	((fs fe)(is cl))	4	32
89	((fs fe)(is fs))	4	43
90	((fs fe)(wa fs))	4	31
91	((fs fs)(cl fe))	4	14

92	((fs hpl) fe)	3	15
93	((fs hpl)(fs fe))	4	12
94	((fs im)(is fs))	4	67
95	((fs is) fe)	3	20
96	((fs is)(fs fe))	4	20
97	((fs is)(wa fs))	4	52
98	((fs wa) fe)	3	19
99	((fs wa)(cl fs))	4	31
100	((fs wa)(fe fs))	4	17
101	((fs wa)((is fs) fe))	5	17
102	((fs wa)(fs hpl))	4	15
103	((fs wa)(is fs))	4	26
104	((im is) fpl)	3	53
105	((im is) im)	3	331
106	((im is)(cl fpl))	4	19
107	((is cl) is)	3	221
108	((is cl)(fe is))	4	32
109	((is cl)(fs im))	4	25
110	((is fs) fe)	3	49
111	((is fs)(fe is))	4	34
112	((is fs)(is fe))	4	19
113	((is fs)(is fpl))	4	27
114	((is fs)(wa is))	4	52
115	((is is)(fs hpl))	4	32
116	((is is)(wa fe))	4	25
117	((is wa) fe)	3	26
118	((is wa)(fe cl))	4	25
119	((is wa)(fs hpl))	4	25
120	((is wa)(fs im))	4	27
121	((is wa)(fs is))	4	72
122	((wa cl) wa)	3	95
123	((wa cl)(fs im))	4	23
124	((wa cl)(is wa))	4	84
125	((wa fs) fe)	3	30
126	((wa fs)(cl wa))	4	49
127	((wa fs)(fe cl))	4	25
128	((wa fs)(fe wa))	4	22
129	((wa fs)(is wa))	4	60
130	((wa is)(cl wa))	4	74
131	((wa is)(fe wa))	4	21
132	((wa is)(fs fe))	4	22
133	((wa is)(fs hpl))	4	19
134	((wa is)(fs wa))	4	33
135	(fe ((cl wa)(fs im)))	5	18
136	(fs (cl (fe cl)))	4	14
137	(fs (fe (is fs)))	4	46
138	(wa (fs (fe cl)))	4	20
139	(wa ((fs fe)(is cl)))	5	16
140	(wa ((is fs) fe))	4	23
141	((cl fs)(fe (is cl)))	5	31

142	((cl fs)((fe is) cl))	5	26
143	((cl is)(fe (is cl)))	5	31
144	((cl is)(fs (fe cl)))	5	27
145	((cl is)(fs (fe is)))	5	25
146	((cl is)((fe is) cl))	5	27
147	((cl is)((fs fe) is))	5	24
148	((cl is)((fs fe)(is cl)))	6	26
149	((cl is)((wa cl)(fs im)))	6	21
150	((cl wa)((cl wa)(fs im)))	6	22
151	((fe is)((cl wa)(fs im)))	6	18
152	((fs cl)((fe is) fs))	5	17
153	((fs (is fs))((cl fe) cl))	6	13
154	((fs fs)((cl fs)(fe cl)))	6	12
155	((fs fs)((cl fs)(fe is)))	6	12
156	((fs hpl)(fe (is fs)))	5	15
157	((fs is)((fs fe)(wa fs)))	6	11
158	((fs wa)(fs (fe fs)))	5	15
159	((fs wa)((fs fe)(is fs)))	6	17
160	((fs wa)((is is)(fs hpl)))	6	14
161	((is cl)(fs (fe is)))	5	27
162	((is cl)((fs fe) is))	5	26
163	((is cl)((is cl)(fs im)))	6	22
164	((is cl)((is fs)(fe is)))	6	25
165	((is cl)((is fs)(is fe)))	6	15
166	((is cl)((wa cl)(fs im)))	6	21
167	((is fe)((cl wa)(fs im)))	6	17
168	((is fs)(cl (fe cl)))	5	15
169	((is fs)((cl fe) cl))	5	16
170	((is fs)((cl fe) is))	5	15
171	((is fs)((cl fe)(is cl)))	6	14
172	((is fs)((is fs) fe))	5	17
173	((is fs)((is fs)(fe is)))	6	14
174	((is is)(fs (cl fe)))	5	15
175	((is is)(fs (is fe)))	5	18
176	((is is)(fs (is fpl)))	5	23
177	((is is)(wa (fs wa)))	5	30
178	((is is)(wa (is fe)))	5	24
179	((is is)((cl fpl) wa))	5	33
180	((is is)((cl wa)(fs im)))	6	25
181	((is is)((fs is) fe))	5	17
182	((is is)((fs is)(fs fe)))	6	17
183	((is is)((wa cl)(fs im)))	6	20
184	((is is)((wa fs) fe))	5	28
185	((is is)((wa fs)(fe cl)))	6	24
186	((is is)((wa is)(fs fe)))	6	20
187	((is wa)(fe (is cl)))	5	22
188	((is wa)(fs (fe cl)))	5	19
189	((is wa)((fs fe)(is cl)))	6	18
190	((is wa)((is fs) fe))	5	21
191	((wa cl)((is is)(fs hpl)))	6	16

192	((wa fs)(fe (is cl)))	5	23
193	((wa fs)((fe is) wa))	5	20
194	((wa fs)((is fs) fe))	5	21
195	((wa is)(fs (fe cl)))	5	20
196	((wa is)(fs (fe fs)))	5	19
197	((wa is)(fs (fe wa)))	5	14
198	((wa is)((fs fe)(is cl)))	6	19
199	((wa wa)((is is)(fs hpl)))	6	9
200	((fe (is fs))(is im))	5	18
201	((fs (cl fe))(cl is))	5	15
202	((fs (cl fe))(cl wa))	5	14
203	((fs (cl fe))(is cl))	5	13
204	((fs (cl fe))((is cl) is))	6	13
205	((fs (cl fe))(is is))	5	14
206	((fs (cl fe))(wa cl))	5	14
207	((fs (cl fe))((wa cl) wa))	6	14
208	((fs (cl fe))((wa cl)(is wa)))	7	13
209	((fs (fe cl))(is fs))	5	30
210	((fs (fe is)) cl)	4	27
211	((fs (fe is))(wa cl))	5	24
212	((fs (fe is))(wa fs))	5	24
213	((fs (is fe))(cl wa))	5	15
214	((fs (is fe))(is fs))	5	19
215	((fs (is fe))(wa cl))	5	15
216	((fs (is fe))(wa is))	5	16
217	((fs (is fe))(wa (cl wa)))	6	15
218	((fs (is fe))(wa (is wa)))	6	15
219	((fs (is fe))((wa is)(cl wa)))	7	14
220	((fs (is fs))(cl (fe cl)))	6	12
221	((fs (is fs))((cl fe) is))	6	12
222	((fs (is fs))((cl fe)(is cl)))	7	11
223	((is (cl is))(fe is))	5	31
224	((is (cl is))(fs (fe is)))	6	26
225	((is (cl is))((fs fe) is))	6	25
226	((is (cl is))((wa cl)(fs im)))	7	21
227	((wa (cl wa))(fs im))	5	22
228	((wa (fs hpl))(is fpl))	5	16
229	((wa (is wa))(fs hpl))	5	20
230	((wa (is wa))(fs (fe fs)))	6	12
231	((wa (is wa))((is is)(fs hpl)))	7	13
232	(((cl fe) is) cl)	4	32
233	(((cl fpl) is)(wa is))	5	40
234	(((cl fpl) wa)(fs im))	5	18
235	(((cl fpl)(is wa))(fs im))	6	16
236	(((cl fs) fe) cl)	4	34
237	(((cl fs) fe)(is cl))	5	30
238	(((cl is) cl)(fs im))	5	23
239	(((cl is)(fe is)) cl)	5	26
240	(((cl wa) cl)((is is)(fs hpl)))	7	14
241	(((fe is)(wa cl))(fs im))	6	16

242	(((fs cl)(is im))(fe is))	6	11
243	(((fs cl)(is im))(wa cl))	6	15
244	(((fs cl)(is im))(wa fs))	6	15
245	(((fs cl)(is im))((fe is) cl))	7	10
246	(((fs cl)(is im))((fe is) fs))	7	10
247	(((fs fe) is) cl)	4	26
248	(((fs fe) is)(wa cl))	5	22
249	(((fs fe) is)(wa fs))	5	22
250	(((fs fe) is)((cl wa) cl))	6	19
251	(((fs fe)(is cl))(is fs))	6	29
252	(((fs fe)(is cl))(wa fs))	6	25
253	(((fs fs)(cl fe))(cl is))	6	12
254	(((fs fs)(cl fe))(cl wa))	6	12
255	(((fs fs)(cl fe))((is cl) is))	7	11
256	(((fs fs)(cl fe))((wa cl) wa))	7	12
257	(((fs hpl) fe)(cl wa))	5	11
258	(((fs is) fe)(is fs))	5	19
259	(((fs is) fe)(wa cl))	5	14
260	(((fs is) fe)(wa is))	5	16
261	(((fs is) fe)(wa (cl wa)))	6	14
262	(((fs is)(fs fe))(cl wa))	6	14
263	(((fs is)(fs fe))(is fs))	6	20
264	(((fs is)(fs fe))(wa cl))	6	14
265	(((fs is)(fs fe))(wa (cl wa)))	7	14
266	(((fs wa)(is fs)) fe)	5	15
267	(((fs wa)(is fs))(fe cl))	6	13
268	(((fs wa)(is fs))(fe fs))	6	13
269	(((fs wa)(is fs))(fe (is cl)))	7	12
270	(((is cl) is)(fe is))	5	29
271	(((is cl) is)(fs (fe is)))	6	24
272	(((is cl) is)((fs fe) is))	6	23
273	(((is cl)(fs im))(is is))	6	23
274	(((is fs)(is fe))(wa is))	6	15
275	(((is fs)(is fpl))(is wa))	6	20
276	(((is is)(fs hpl))(is fpl))	6	19
277	(((is wa)(fs hpl))(is is))	6	21
278	(((is wa)(fs is))(fe fs))	6	14
279	(((is wa)(fs is))(fs fe))	6	23
280	(((is wa)(fs is))(fs (fe cl)))	7	19
281	(((is wa)(fs is))(fs (fe fs)))	7	20
282	(((is wa)(fs is))(fs (fe is)))	7	20
283	(((is wa)(fs is))((fs fe) is))	7	19
284	(((is wa)(fs is))((fs fe)(is cl)))	8	18
285	(((wa cl) wa)(fs im))	5	25
286	(((wa fs) fe) cl)	4	26
287	(((wa fs) fe)(fs fs))	5	13
288	(((wa fs) fe)((is cl) is))	6	23
289	(((wa fs) fe)(is is))	5	27
290	(((wa fs) fe)(is (cl is)))	6	22
291	(((wa is)(fs fe)) cl)	5	21

292	(((wa is)(fs fe))(fs is))	6	21
293	(((wa is)(fs fe))((is is)(fs hpl)))	8	15
294	(fe ((wa (cl wa))(fs im)))	6	16
295	(fe (((wa cl) wa)(fs im)))	6	18
296	((cl is)((fs (fe is)) cl))	6	21
297	((cl is)(((fs fe) is) cl))	6	22
298	((cl is)(((fs is)(fs fe))(cl wa)))	8	13
299	((cl is)(((fs is)(fs fe))(wa cl)))	8	13
300	((cl is)(((fs is)(fs fe))(wa (cl wa))))	9	13
301	((fe cl)((wa (cl wa))(fs im)))	7	15
302	((fe is)((wa (cl wa))(fs im)))	7	16
303	((fe is)(((wa cl) wa)(fs im)))	7	18
304	((fs cl)((fs (fe is))(wa cl)))	7	18
305	((fs cl)(((fs is)(fs fe))(cl wa)))	8	13
306	((fs cl)(((fs is)(fs fe))(wa cl)))	8	13
307	((fs cl)(((fs is)(fs fe))(wa (cl wa))))	9	13
308	((fs fs)((fe (is fs))(is im)))	7	16
309	((fs fs)(((cl fs) fe)(is cl)))	7	11
310	((fs hpl)(fs (fe (is fs))))	6	13
311	((((fs is)(fs fe))(is fs))(is im))	8	11
312	((fs is)(((fs fs)(cl fe))(cl wa)))	8	11
313	((fs is)(((fs fs)(cl fe))((wa cl) wa)))	9	11
314	((is cl)((is fs)((is fs) fe)))	7	15
315	((is cl)((is fs)((is fs)(fe is))))	8	13
316	((is cl)((is is)(fs (is fe))))	7	13
317	((is cl)((is is)((cl wa)(fs im))))	8	23
318	((is cl)((is is)((fs is) fe)))	7	12
319	((is cl)((is is)((fs is)(fs fe))))	8	13
320	((is cl)(((fs is)(fs fe))(cl wa)))	8	13
321	((is cl)(((fs is)(fs fe))(wa cl)))	8	13
322	((is cl)(((fs is)(fs fe))(wa (cl wa))))	9	13
323	((is cl)(((is cl)(fs im))(is is)))	8	21
324	((is fe)((wa (cl wa))(fs im)))	7	15
325	((is fe)(((wa cl) wa)(fs im)))	7	16
326	((is fs)(fe ((cl wa)(fs im))))	7	16
327	((is fs)((fe (is fs))(is im)))	7	16
328	((is fs)(((fe is)(wa cl))(fs im)))	8	14
329	((is is)(fs (cl (fe cl))))	6	13
330	((is is)(wa (fs (fe cl))))	6	19
331	((is is)(wa ((fs fe)(is cl))))	7	15
332	((is is)((cl is)((wa cl)(fs im))))	8	19
333	((is is)((fs (is fs))((cl fe) cl)))	8	12
334	((is is)((fs fs)((cl fs)(fe cl))))	8	11
335	((is is)((wa fs)((is fs) fe)))	7	20
336	((is is)((wa is)(fs (fe cl))))	7	19
337	((is is)((wa is)(fs (fe fs))))	7	18
338	((is is)((fs (cl fe))(cl is)))	7	14
339	((is is)((fs (cl fe))(cl wa)))	7	13
340	((is is)((fs (cl fe))(wa cl)))	7	13
341	((is is)((fs (cl fe))((wa cl) wa)))	8	13

342	((is is)((fs (cl fe))(is cl)))	7	12
343	((is is)((fs (cl fe))((is cl) is)))	8	12
344	((is is)((fs (cl fe))(is is)))	7	13
345	((is is)((fs (cl fe))((wa cl)(is wa))))	9	12
346	((is is)((fs (is fs))(cl (fe cl))))	8	11
347	((is is)(((fs fs)(cl fe))(cl is)))	8	11
348	((is is)((wa (cl wa))(fs im)))	7	20
349	((is is)((wa (fs hpl))(is fpl)))	7	15
350	((is is)((wa (is wa))(fs hpl)))	7	18
351	((is is)((wa (is wa))(fs (fe fs))))	8	9
352	((is is)(((cl fpl) wa)(fs im)))	7	15
353	((is is)(((cl fpl)(is wa))(fs im)))	8	14
354	((is is)(((cl wa) cl)((is is)(fs hpl))))	9	11
355	((is is)(((fs cl)(is im))(fe is)))	8	10
356	((is is)(((fs cl)(is im))((fe is) cl)))	9	9
357	((is is)(((fs cl)(is im))((fe is) fs)))	9	9
358	((is is)(((fs fs)(cl fe))(cl wa)))	8	11
359	((is is)(((wa fs) fe) cl))	6	25
360	((is is)(((wa fs) fe)(cl is)))	7	24
361	((is is)(((wa fs) fe)(is is)))	7	25
362	((is is)(((wa fs)(fe cl))(is is)))	8	22
363	((is is)(((wa is)(fs fe)) cl))	7	20
364	((is is)(((wa is)(fs fe))(fs is)))	8	20
365	((is is)(((wa is)(fs fe))(cl is)))	8	19
366	((is wa)((fs (is fe))(is fs)))	7	11
367	((wa is)((wa fs)((is fs) fe)))	7	18
368	((fs (cl fs))((fe (is fs))(is im)))	8	15
369	((fs (fe is))(((cl fpl) is)(wa is)))	8	17
370	((fs (is fs))((fe (is fs))(is im)))	8	16
371	((is (cl is))((wa (cl wa))(fs im)))	8	19
372	((is (cl is))(((fs is)(fs fe))(cl wa)))	9	13
373	((is (cl is))(((fs is)(fs fe))(wa cl)))	9	13
374	((is (cl is))(((fs is)(fs fe))(wa (cl wa))))	10	13
375	((wa (is wa))((is fs)((is fs) fe)))	8	10
376	(((cl fe) cl)((wa (cl wa))(fs im)))	8	14
377	(((fs cl) fs)((fe (is fs))(is im)))	8	14
378	(((fs cl)(is fs))((fe (is fs))(is im)))	9	13
379	(((is wa)(fs hpl))(fs (fe (is fs))))	8	12
380	(((is wa)(fs is))((fs (fe is)) cl))	8	17
381	((fs (cl (fe cl)))(fs fs))	6	14
382	((fs (cl (fe cl)))(is fs))	6	14
383	((fs (cl (fe cl)))(is is))	6	14
384	((fs (fe (is fs)))(is im))	6	14
385	(((cl is)(fs (fe cl)))(is fpl))	7	19
386	(((fs (is fs))((cl fe) cl))(is fs))	8	13
387	(((fs (is fs))((cl fe) cl))(is is))	8	13
388	(((fs (is fs))((cl fe) cl))((wa cl)(is wa)))	10	10
389	(((fs fs)((cl fs)(fe cl)))(is fs))	8	12
390	(((fs (is fs))(cl (fe cl)))(is fs))	8	12
391	(((fs fs)((cl fs)(fe cl)))(is is))	8	12

392	(((fs (is fs))(cl (fe cl)))(is is))	8	12
393	(((is cl)(fs (fe is)))(((cl fpl) is)(wa is)))	10	17
394	(((is cl)((is fs)(is fe)))(cl wa))	8	13
395	(((is cl)((is fs)(is fe)))(wa cl))	8	13
396	(((is cl)((is fs)(is fe)))(wa (cl wa)))	9	13
397	(((is fs)(cl (fe cl)))(fs is))	7	14
398	(((is fs)(cl (fe cl)))(is fs))	7	15
399	(((is fs)(cl (fe cl)))(is is))	7	15
400	(((is fs)((cl fe) cl))(fs is))	7	15
401	(((is fs)((cl fe) cl))(is fs))	7	16
402	(((is fs)((cl fe) cl))(is is))	7	16
403	(((is fs)((cl fe) cl))(wa cl))	7	13
404	(((is fs)((cl fe) cl))((is fs)(wa is)))	9	13
405	(((is fs)((cl fe) cl))((wa cl) wa))	8	13
406	(((is fs)((cl fe) cl))((wa cl)(is wa)))	9	13
407	(((is fs)((cl fe) is))(cl fs))	7	14
408	(((is fs)((cl fe) is))((wa cl)(is wa)))	9	13
409	(((is fs)((cl fe)(is cl)))(is fs))	8	14
410	(((is fs)((cl fe)(is cl)))(is is))	8	13
411	(((is fs)((is fs) fe))(cl wa))	7	14
412	(((is is)((fs is)(fs fe)))(cl wa))	8	14
413	(((is fs)((is fs) fe))(wa (cl wa)))	8	14
414	(((is is)((fs is)(fs fe)))(wa (cl wa)))	9	14
415	(((is is)(fs (is fe)))(cl is))	7	16
416	(((is is)(fs (is fe)))(cl wa))	7	15
417	(((is is)(fs (is fe)))(is is))	7	16
418	(((is is)(fs (is fe)))(wa cl))	7	15
419	(((is is)(fs (is fe)))(wa (cl wa)))	8	15
420	(((is is)(wa (is fe)))(cl is))	7	22
421	(((is is)(wa (is fe)))(fs is))	7	20
422	(((is is)((cl wa)(fs im)))(is is))	8	23
423	(((is is)((fs is) fe))(cl wa))	7	14
424	(((is is)((fs is) fe))(wa cl))	7	14
425	(((is is)((fs is) fe))(wa (cl wa)))	8	14
426	(((is is)((wa fs) fe))(fs fs))	7	12
427	(((is is)((wa fs) fe))(is is))	7	23
428	(((is is)((wa is)(fs fe)))(fs is))	8	17
429	(((is wa)((is fs) fe))(cl is))	7	21
430	(((is wa)((is fs) fe))(fs is))	7	17
431	(((wa fs)((is fs) fe))(fs is))	7	21
432	(((wa fs)((is fs) fe))(is fs))	7	21
433	(((wa fs)((is fs) fe))(is is))	7	20
434	(((wa is)(fs (fe fs)))(is cl))	7	17
435	(((wa is)(fs (fe fs)))((is cl) is))	8	17
436	(((wa is)(fs (fe fs)))(is is))	7	19
437	(((fs (cl fe))(cl is))(cl wa))	7	13
438	(((fs (cl fe))(cl is))(fs fs))	7	15
439	(((fs (cl fe))(cl is))(fs is))	7	14
440	(((fs (cl fe))(cl is))(wa cl))	7	12
441	(((fs (cl fe))(cl is))((cl wa) cl))	8	12

442	(((fs (cl fe))(cl is))((is fs)(wa is)))	9	12
443	(((fs (cl fe))(cl is))((wa cl) wa))	8	12
444	(((fs (cl fe))(cl is))((wa cl)(is wa)))	9	12
445	(((fs (cl fe))(cl wa))(cl cl))	7	11
446	(((fs (cl fe))(cl wa))(cl is))	7	11
447	(((fs (cl fe))(cl wa))(is cl))	7	11
448	(((fs (cl fe))(cl wa))((is cl) is))	8	11
449	(((fs (cl fe))(cl wa))((cl is) cl))	8	11
450	(((fs (cl fe))((is cl) is))(fs fs))	8	13
451	(((fs (cl fe))(is cl))(is fs))	7	13
452	(((fs (cl fe))(is cl))(is is))	7	12
453	(((fs (cl fe))(wa cl))(wa is))	7	13
454	(((fs (cl fe))(wa cl))(is (cl is)))	8	10
455	(((fs (cl fe))((wa cl) wa))(im is))	8	11
456	(((fs (fe is)) cl)(is fs))	6	24
457	(((fs (fe is)) cl)(wa fs))	6	22
458	(((fs (fe is))(wa cl))(is fs))	7	22
459	(((fs (fe is))(wa cl))(wa fs))	7	20
460	(((fs (is fe))(cl wa))(fs is))	7	12
461	(((fs (is fe))(cl wa))(is fs))	7	14
462	(((fs (is fe))(wa cl))(fs is))	7	13
463	(((fs (is fe))(wa cl))(is fs))	7	14
464	(((fs (is fe))(wa cl))(is is))	7	15
465	(((fs (is fe))(wa cl))(wa fs))	7	13
466	(((fs (is fe))(wa cl))(wa is))	7	15
467	(((fs (is fe))(wa is))(cl is))	7	15
468	(((fs (is fe))(wa is))(wa is))	7	15
469	(((fs (is fe))(wa (cl wa)))(im is))	8	12
470	(((fs (is fe))(wa (cl wa)))(is fs))	8	12
471	(((fs (is fs))((cl fe)(is cl)))(is is))	9	10
472	(((wa (is wa))(fs (fe fs)))(cl is))	8	11
473	(((wa (is wa))(fs (fe fs)))(is cl))	8	11
474	(((wa (is wa))(fs (fe fs)))((is cl) is))	9	11
475	(((wa (is wa))(fs (fe fs)))(is fpl))	8	11
476	((((cl fpl) wa)(fs im))(is is))	7	18
477	((((cl fpl)(is wa))(fs im))(is is))	8	16
478	((((fs fe) is) cl)(is fs))	6	23
479	((((fs fe) is)(wa cl))(is fs))	7	20
480	((((fs fe) is)(wa cl))(is im))	7	12
481	((((fs fe) is)(wa cl))(wa fs))	7	19
482	((((fs fs)(cl fe))(cl wa))(cl cl))	8	9
483	((((fs fs)(cl fe))(cl wa))(cl is))	8	9
484	((((fs fs)(cl fe))(cl wa))(is cl))	8	9
485	((((fs fs)(cl fe))(cl wa))((is cl) is))	9	9
486	((((fs fs)(cl fe))(cl wa))((cl is) cl))	9	9
487	((((fs fs)(cl fe))((wa cl) wa))(im is))	9	8
488	((((fs hpl) fe)(cl wa))(fs is))	7	10
489	((((fs hpl) fe)(cl wa))(is fs))	7	10
490	((((fs is) fe)(is fs))(is im))	7	10
491	((((fs is) fe)(wa cl))(fs is))	7	12

492	((((fs is) fe)(wa cl))(is fs))	7	13
493	((((fs is) fe)(wa cl))(is is))	7	13
494	((((fs is) fe)(wa cl))(wa fs))	7	12
495	((((fs is) fe)(wa cl))(wa is))	7	14
496	((((fs is) fe)(wa is))(cl is))	7	14
497	((((fs is) fe)(wa is))(cl wa))	7	13
498	((((fs is) fe)(wa is))(wa is))	7	14
499	((((fs is) fe)(wa (cl wa)))(im is))	8	10
500	((((fs is) fe)(wa (cl wa)))(is fs))	8	11
501	((((fs is)(fs fe))(cl wa))(fs is))	8	11
502	((((fs is)(fs fe))(cl wa))(is fs))	8	13
503	((((fs is)(fs fe))(wa cl))(fs is))	8	12
504	((((fs is)(fs fe))(wa cl))(is fs))	8	13
505	((((fs is)(fs fe))(wa cl))(is is))	8	13
506	((((fs is)(fs fe))(wa cl))(wa fs))	8	12
507	((((fs is)(fs fe))(wa cl))(wa is))	8	14
508	((((fs is)(fs fe))(wa (cl wa)))(is fs))	9	11
509	((((fs wa)(is fs)) fe)((is cl) is))	8	12
510	((((fs wa)(is fs)) fe)((is is)(fs hpl)))	9	11
511	((((fs wa)(is fs))(fe cl))(is fs))	8	13
512	((((fs wa)(is fs))(fe cl))((is is)(fs hpl)))	10	10
513	((((fs wa)(is fs))(fe fs))(is cl))	8	11
514	((((fs wa)(is fs))(fe fs))((is cl) is))	9	11
515	((((is wa)(fs hpl))(is is))(cl is))	8	14
516	((((is wa)(fs hpl))(is is))(fs is))	8	11
517	((((is wa)(fs is))(fe fs))(cl is))	8	12
518	((((is wa)(fs is))(fe fs))(is cl))	8	12
519	((((is wa)(fs is))(fe fs))((is is)(fs hpl)))	10	11
520	((((is wa)(fs is))(fs fe))(cl is))	8	18
521	((((is wa)(fs is))(fs fe))(is cl))	8	17
522	((((is wa)(fs is))(fs fe))((is cl) is))	9	17
523	((((is wa)(fs is))(fs (fe fs)))(cl is))	9	15
524	((((is wa)(fs is))(fs (fe fs)))(is cl))	9	15
525	((((is wa)(fs is))(fs (fe fs)))((is cl) is))	10	15
526	((((wa fs) fe)(is is))(cl is))	7	21
527	((cl is)((((fs is)(fs fe))(wa cl))(is is)))	10	12
528	((cl is)((((fs is)(fs fe))(wa cl))(wa is)))	10	13
529	((fs cl)((fs fs)((fe (is fs))(is im))))	9	13
530	((fs cl)((fs (is fs))((fe (is fs))(is im))))	10	13
531	((fs cl)(((is fs)((is fs) fe))(cl wa)))	9	13
532	((fs cl)(((is fs)((is fs) fe))(wa (cl wa))))	10	13
533	((fs cl)(((is is)((fs is)(fs fe)))(cl wa)))	10	11
534	((fs cl)(((is is)((fs is)(fs fe)))(wa (cl wa))))	11	11
535	((fs cl)(((is is)(fs (is fe)))(wa cl)))	9	11
536	((fs cl)(((fs (is fe))(wa (cl wa)))(im is)))	10	11
537	((fs cl)((((fs is)(fs fe))(wa cl))(fs im)))	10	13
538	((fs cl)((((fs is)(fs fe))(wa cl))(is is)))	10	12
539	((fs cl)((((fs is)(fs fe))(wa cl))(wa is)))	10	13
540	((fs cl)((((fs is)(fs fe))(wa (cl wa)))(im is)))	11	10
541	((fs fs)((is fs)((fe (is fs))(is im))))	9	10

542	((fs is)((fs (fe (is fs)))(is im)))	8	9
543	((is cl)((fe cl)((wa (cl wa))(fs im))))	9	13
544	((is cl)(((is fs)((is fs) fe))(cl wa)))	9	13
545	((is cl)(((is fs)((is fs) fe))(wa (cl wa))))	10	13
546	((is cl)(((is is)((fs is)(fs fe)))(cl wa)))	10	11
547	((is cl)(((is is)((fs is)(fs fe)))(wa (cl wa))))	11	11
548	((is cl)(((is is)(fs (is fe)))(cl wa)))	9	11
549	((is cl)(((is is)(fs (is fe)))(wa cl)))	9	11
550	((is cl)(((is is)(fs (is fe)))(wa (cl wa))))	10	11
551	((is cl)(((is is)((cl wa)(fs im)))(is is)))	10	22
552	((is cl)((((fs is)(fs fe))(wa cl))(is is)))	10	12
553	((is cl)((((fs is)(fs fe))(wa cl))(wa is)))	10	13
554	((is is)((fs cl)((fs (fe is))(wa cl))))	9	17
555	((is is)((fs (cl (fe cl)))(is is)))	8	13
556	((is is)((fs (fe (is fs)))(is im)))	8	14
557	((is is)((wa ((is fs) fe))(cl is)))	8	20
558	((is is)(((fs (is fs))((cl fe) cl))((wa cl)(is wa))))	12	9
559	((is is)(((wa fs)(fe (is cl)))(is is)))	9	19
560	((is is)(((wa fs)((is fs) fe))(fs is)))	9	20
561	((is is)(((wa fs)((is fs) fe))(is fs)))	9	20
562	((is is)(((wa fs)((is fs) fe))(is is)))	9	19
563	((is is)(((wa is)(fs (fe fs)))(cl is)))	9	16
564	((is is)(((wa is)(fs (fe fs)))(is (cl is))))	10	16
565	((is is)(((wa is)(fs (fe fs)))(is cl)))	9	16
566	((is is)(((wa is)(fs (fe fs)))(is is)))	9	18
567	((is is)(((fs (cl fe))(cl is))(cl wa)))	9	12
568	((is is)(((fs (cl fe))(cl is))(wa cl)))	9	11
569	((is is)(((fs (cl fe))(cl is))((cl wa) cl)))	10	11
570	((is is)(((fs (cl fe))(cl is))((wa cl) wa)))	10	11
571	((is is)(((fs (cl fe))(cl is))((wa cl)(is wa))))	11	11
572	((is is)(((fs (cl fe))(cl is))((is fs)(wa is))))	11	11
573	((is is)(((fs (cl fe))(cl wa))(cl cl)))	9	10
574	((is is)(((fs (cl fe))(cl wa))(cl is)))	9	10
575	((is is)(((fs (cl fe))(cl wa))(is cl)))	9	10
576	((is is)(((fs (cl fe))(cl wa))((is cl) is)))	10	10
577	((is is)(((fs (cl fe))(cl wa))((cl is) cl)))	10	10
578	((is is)(((fs (cl fe))(is cl))(is is)))	9	11
579	((is is)(((fs (cl fe))(wa cl))(is is)))	9	12
580	((is is)(((fs (cl fe))(wa cl))(wa is)))	9	12
581	((is is)(((fs (cl fe))((wa cl) wa))(im is)))	10	10
582	((is is)((((cl fpl) wa)(fs im))(is is)))	9	15
583	((is is)((((cl fpl)(is wa))(fs im))(is is)))	10	14
584	((is is)((((fs fe) is)(wa cl))(is fs)))	9	17
585	((is is)((((wa fs) fe) cl)(is is)))	8	22
586	((is is)((((wa is)(fs fe)) cl)(is is)))	9	19
587	((is is)((((wa is)(fs fe))(fs is))(cl is)))	10	17
588	((wa is)(((wa fs)((is fs) fe))(fs is)))	9	18
589	((wa is)(((wa fs)((is fs) fe))(is fs)))	9	18
590	((wa is)(((wa fs)((is fs) fe))(is is)))	9	17
591	((is (cl is))((((fs is)(fs fe))(wa cl))(is is)))	11	12

592	((is (cl is))((((fs is)(fs fe))(wa cl))(wa is)))	11	13
593	(((is wa)(fs is))((fs fs)((fe (is fs))(is im))))	11	12
594	((((is cl)((is fs)(is fe)))(wa cl))(is is))	10	12
595	((((is cl)((is fs)(is fe)))(wa cl))(wa is))	10	13
596	(((cl is)(((fs is)(fs fe))(wa (cl wa))))(im is))	11	11
597	(((fs cl)(((fs is)(fs fe))(wa (cl wa))))(im is))	11	11
598	(((is cl)(((fs is)(fs fe))(wa (cl wa))))(im is))	11	11
599	(((is (cl is))(((fs is)(fs fe))(wa (cl wa))))(im is))	12	11
600	((((is cl)((is fs)(is fe)))(wa (cl wa)))(im is))	11	11
601	(((fs cl)((fs (fe is))(wa cl)))(is cl))	9	14
602	(((fs cl)((fs (fe is))(wa cl)))((is cl) is))	10	14
603	(((fs cl)((fs (fe is))(wa cl)))(wa is))	9	18
604	(((fs fs)(((cl fs) fe) cl))(is is))	8	14
605	(((fs fs)(((cl fs) fe)(is cl)))(is is))	9	10
606	(((fs is)(((fs fs)(cl fe))((wa cl) wa)))(im is))	11	8
607	(((is cl)((is fs)((is fs) fe)))(wa cl))	9	13
608	(((is is)(wa ((is fs) fe)))(cl is))	8	21
609	(((fs (cl (fe cl)))(is is))(fs is))	8	13
610	((((is fs)(cl (fe cl)))(fs is))(wa (is wa)))	10	12
611	((((is fs)(cl (fe cl)))(is is))(fs is))	9	14
612	((((is fs)((cl fe) cl))(is is))(fs fs))	9	15
613	((((is fs)((cl fe) cl))(is is))(fs is))	9	15
614	((((is fs)((cl fe) cl))(wa cl))(im is))	9	12
615	((((is fs)((cl fe) cl))(wa cl))(is cl))	9	12
616	((((is fs)((cl fe) cl))(wa cl))((is cl) is))	10	12
617	((((is fs)((cl fe) cl))(wa cl))(is is))	9	10
618	((((is fs)((is fs) fe))(cl wa))(fs is))	9	11
619	((((is is)((fs is)(fs fe)))(cl wa))(fs is))	10	11
620	((((is fs)((is fs) fe))(cl wa))(is fs))	9	13
621	((((is is)((fs is)(fs fe)))(cl wa))(is fs))	10	13
622	((((is is)((fs is)(fs fe)))(wa (cl wa)))(im is))	11	10
623	((((is fs)((is fs) fe))(wa (cl wa)))(is fs))	10	11
624	((((is is)((fs is)(fs fe)))(wa (cl wa)))(is fs))	11	11
625	((((is is)(fs (is fe)))(cl wa))(fs is))	9	12
626	((((is is)(fs (is fe)))(cl wa))(is fs))	9	14
627	((((is is)(fs (is fe)))(wa cl))(fs is))	9	13
628	((((is is)(fs (is fe)))(wa cl))(is fs))	9	14
629	((((is is)(fs (is fe)))(wa cl))(is is))	9	15
630	((((is is)(fs (is fe)))(wa cl))(wa fs))	9	13
631	((((is is)(fs (is fe)))(wa cl))(wa is))	9	15
632	((((is is)(fs (is fe)))(wa (cl wa)))(im is))	10	12
633	((((is is)(fs (is fe)))(wa (cl wa)))(is fs))	10	12
634	((((is is)(wa (is fe)))(fs is))((is is)(fs hpl)))	11	10
635	((((is is)((cl fpl) wa))(fs im))(is is))	9	17
636	((((is is)((fs is) fe))(cl wa))(is fs))	9	13
637	((((is is)((fs is) fe))(wa cl))(fs is))	9	12
638	((((is is)((fs is) fe))(wa cl))(is fs))	9	13
639	((((is is)((fs is) fe))(wa cl))(is is))	9	13
640	((((is is)((fs is) fe))(wa cl))(wa fs))	9	12
641	((((is is)((fs is) fe))(wa cl))(wa is))	9	14

642	((((is is)((fs is) fe))(wa (cl wa)))(im is))	10	10
643	((((is is)((fs is) fe))(wa (cl wa)))(is fs))	10	11
644	((((is is)((wa is)(fs fe)))(fs is))(cl is))	10	15
645	((((is wa)((is fs) fe))(fs is))(cl is))	9	15
646	((((is is)((wa is)(fs fe)))(fs is))(is fpl))	10	14
647	((((is wa)((is fs) fe))(fs is))(is fpl))	9	14
648	((((is is)((wa is)(fs fe)))(fs is))((is is)(fs hpl)))	12	9
649	((((is wa)((is fs) fe))(fs is))((is is)(fs hpl)))	11	9
650	((((fs (cl fe))(cl is))(cl wa))(cl cl))	9	11
651	((((fs (cl fe))(cl is))(cl wa))(is cl))	9	11
652	((((fs (cl fe))(cl is))(cl wa))((is cl) is))	10	11
653	((((fs (cl fe))(cl is))(cl wa))(is is))	9	13
654	((((fs (cl fe))(cl is))(cl wa))((cl is) cl))	10	11
655	((((fs (cl fe))(cl is))(wa cl))(is cl))	9	11
656	((((fs (cl fe))(cl is))(wa cl))((is cl) is))	10	11
657	((((fs (cl fe))(cl is))((cl wa) cl))(is cl))	10	12
658	((((fs (cl fe))(cl is))((cl wa) cl))((is cl) is))	11	12
659	((((fs (cl fe))(cl is))((cl wa) cl))(is is))	10	11
660	((((fs (cl fe))(cl wa))(cl cl))(is fpl))	9	10
661	((((fs (cl fe))(cl wa))(cl is))(cl is))	9	10
662	(((((fs wa)(is fs))(fe cl))(is is))(wa fs))	10	12
663	(((((fs wa)(is fs)) fe)(cl is))((is wa)(fs is)))	11	12
664	(((((is wa)(fs hpl))(is is))(cl is))(cl wa))	10	13
665	(((((is wa)(fs is))(fe fs))((is cl) is))(wa fs))	11	12
666	(((((is wa)(fs is))(fe fs))((is cl) is))((is wa)(fs is)))	13	12
667	((fs cl)((((is fs)((is fs) fe))(wa (cl wa)))(im is)))	12	10
668	((fs cl)((((is is)((fs is)(fs fe)))(wa (cl wa)))(im is)))	13	8
669	((fs cl)((((is is)(fs (is fe)))(wa cl))(is is)))	11	11
670	((fs cl)((((is is)(fs (is fe)))(wa cl))(wa is)))	11	11
671	((fs cl)((((is is)(fs (is fe)))(wa (cl wa)))(im is)))	12	9
672	((is cl)((((is is)(fs (is fe)))(wa cl))(is is)))	11	11
673	((is cl)((((is is)(fs (is fe)))(wa cl))(wa is)))	11	11
674	((is is)((fs cl)((((fs is)(fs fe))(wa cl))(is is))))	12	11
675	((is is)(((fs cl)((fs (fe is))(wa cl)))(is cl)))	11	13
676	((is is)(((fs cl)((fs (fe is))(wa cl)))((is cl) is)))	12	13
677	((is is)(((fs cl)((fs (fe is))(wa cl)))(is is)))	11	16
678	((is is)(((fs cl)((fs (fe is))(wa cl)))(wa is)))	11	17
679	((is is)((((wa is)(fs (fe fs)))(is is))(cl is)))	11	15
680	((is is)((((fs (cl fe))(cl is))(cl wa))(is is)))	11	12
681	((is is)((((fs (cl fe))(cl is))(wa cl))(is is)))	11	10
682	((is is)((((fs (cl fe))(cl is))((cl wa) cl))(is cl)))	12	11
683	((is is)((((fs (cl fe))(cl is))((cl wa) cl))((is cl) is)))	13	11
684	((is is)((((fs (cl fe))(cl is))((cl wa) cl))(is is)))	12	10
685	((is is)((((fs (cl fe))(cl wa))(cl cl))(is fpl)))	11	9
686	((fs (is fs))((((is fs)((cl fe) cl))(wa cl))(is is)))	12	9
687	((wa (is wa))((((is fs)((is fs) fe))(cl wa))(is fs)))	12	10
688	(((fs cl)(((is fs)((is fs) fe))(wa (cl wa))))(im is))	12	11
689	(((is cl)(((is fs)((is fs) fe))(wa (cl wa))))(im is))	12	11
690	(((is cl)(((is is)((fs is)(fs fe)))(wa (cl wa))))(im is))	13	8
691	(((is cl)(((is is)(fs (is fe)))(wa (cl wa))))(im is))	12	8

692	(((is is)((fs cl)((fs (fe is))(wa cl))))(wa is))	11	15
693	(((is is)(((fs (cl fe))(cl is))((is fs)(wa is))))(wa wa))	13	9
694	(((wa is)(((wa fs)((is fs) fe))(is is)))(cl wa))	11	15
695	((((is cl)((is fs)((is fs) fe)))(wa cl))(is is))	11	12
696	((((is cl)((is fs)((is fs) fe)))(wa cl))(wa is))	11	13
697	((((is wa)((is fs)((is fs) fe)))(cl is))(fs is))	11	11
698	((((is wa)((is fs)((is fs) fe)))(cl is))(wa fs))	11	11
699	(((((is fs)(cl (fe cl)))(is is))(fs fs))(is is))	11	14
700	(((((fs (cl fe))(cl is))(cl wa))(cl is))(cl is))	11	11
701	((((((is wa)(fs hpl))(is is))(cl is))(cl wa))(fs is))	12	10
702	((((((is wa)(fs hpl))(is is))(cl is))(cl wa))(is wa))	12	11
703	(((is is)((((fs (cl fe))(cl is))((cl wa) cl))(is cl)))(is fpl))	14	10

Total **20148**
